# Compliance with clinical guidelines for whiplash improved with a targeted implementation strategy: a prospective cohort study

**DOI:** 10.1186/1472-6963-13-213

**Published:** 2013-06-13

**Authors:** Trudy Rebbeck, Luciana G Macedo, Christopher G Maher

**Affiliations:** 1 Discipline of Physiotherapy, Faculty of Health Science, The University of Sydney, PO Box 170, Sydney, Lidcombe NSW 1825, Australia; 2 Common Spinal Disorders Research Group, Department of Physical Therapy, Faculty of Rehabilitation Medicine, University of Alberta, 2-50 Corbett Hall, Edmonton, AB T6G 2G4, Canada; 3 The George Institute for Global Health, Sydney Medical School, The University of Sydney, Level 13, 321 Kent St, Sydney NSW 2000, Australia

**Keywords:** Implementation, Interactive education, Clinical guidelines, Whiplash

## Abstract

**Background:**

Implementation strategies for clinical guidelines have shown modest effects in changing health professional’s knowledge and practice, however, targeted implementations are suggested to achieve greater improvements. This study aimed to examine the effect of a targeted implementation strategy of the Australian whiplash guidelines on health professionals’ knowledge, beliefs and practice and to identify predictors of improved knowledge.

**Methods:**

94 health professionals (Physiotherapists, Chiropractors and Osteopaths) who manage whiplash participated in this study. Prior to their inclusion in the study, health professionals were classified as compliant with clinical guidelines for whiplash (n = 52) or non-compliant (n = 42), according to a record of clinical practice. All participants completed a 2- day interactive workshop with outcomes measured at baseline and 3 months following the workshop. The workshop was delivered by opinion leaders, with the educational content focused on the pre-identified knowledge and practice gaps in relation to clinical guidelines for whiplash. Knowledge and health professional beliefs were assessed by a questionnaire and professional practice by record of clinical practice.

**Results:**

Participants significantly increased knowledge (p < 0.0001) and were more likely to be compliant with the guidelines at follow-up (compliant at baseline 58%, follow-up 79%, p = 0.002). Health professional belief systems significantly changed to be more behavioural (p = 0.02) and less biomedical (p = 0.000). Predictors of improved knowledge were baseline knowledge (parameter estimate = -0.6, p = 0.000) and profession (parameter estimate = -3.8, p = 0.003) (adj R^2^ = 35%).

**Conclusions:**

A targeted implementation strategy improved health professional’s knowledge and clinical practice so that they became more compliant with clinical guidelines for whiplash. In addition health professionals’ belief systems significantly changed to be more behavioural in orientation. Baseline knowledge and profession predicted 35% of the variance in improved knowledge.

## Background

Clinical guidelines for whiplash were developed in Australia [1,2] for the purpose of improving professional practice, and ultimately changing health outcomes for people with whiplash. Allied health professionals such as physiotherapists, chiropractors and osteopaths were one of the key targets for implementation of the Australian whiplash guidelines, because they are the most commonly consulted health practitioners for the management of whiplash. It is widely reported that dissemination of clinical guidelines alone is unlikely to change health professional’s knowledge or practice [3]. Rather it is suggested that targeted and active implementation strategies need to be used, and barriers to implementation identified, in order to change both professional knowledge and practice.

Active implementation strategies have been effective in changing health professional’s knowledge and practice, however only moderate effects are reported. For example, the most successful implementation strategies identified in Cochrane systematic reviews have reported an absolute risk difference of 6% (95% CI 1.8-15.9) for improving use of endorsed professional practices with a continuing education intervention [4] and 12% (CI 6-14.5) for the use of opinion leaders [5]. To date there is only one systematic review that has examined the effect of active implementation strategies amongst allied health professionals including physiotherapists and chiropractors [6]. This systematic review reported similarly modest effects [6] suggesting room for improvement. Given this, it appears an examination of factors that may improve guideline adherence is warranted.

To date only a few studies have examined barriers to implementation of guidelines amongst health professionals such as physiotherapists and chiropractors. The beliefs of health professionals have been suggested as a factor that would influence behaviour change. Most whiplash guidelines [7-10], including the Australian guidelines [1,2] advocate for an active and behavioural approach to management. It has been suggested that when health professionals have a strong biomedical belief system, health practices are less likely to change to align with a more behavioural approach, such as those endorsed in the guidelines [11-14]. Furthermore, professional background may explain the differences in these belief systems [13]. Professional background and the belief system of the health professional are therefore potential factors that may arise as barriers to implementation.

Other factors have been identified as effect modifiers to compliance with guidelines amongst allied health professionals. These include the experience of the health professional [15], whether the implementation strategy focuses on changing simple or complex behaviours [4,16], perceived advantage of using the guideline [17] and compatibility between current practice and recommendations [14]. In our previous cluster randomised controlled trial (RCT) [18] implementing guidelines for whiplash, we found that both knowledge and clinical practice were largely consistent with guideline recommendations at baseline, with most physiotherapists already using active treatments. We hypothesised, therefore, that if health professional knowledge and practice were less compliant with guidelines at baseline, the effect on learning should be improved. In order to test this hypothesis, it would be necessary to include a sample of participants whose knowledge and practice was identified as non-compliant with guidelines at baseline.

The primary aim of this study was therefore, to describe and evaluate the effect of a targeted implementation strategy on the knowledge, practice and beliefs of allied health professionals managing patients with whiplash. In addition, this study aimed to identify factors that predicted learning in relation to clinical guidelines for whiplash.

## Methods

### Study design and setting

We conducted a prospective longitudinal study to evaluate the outcomes from a targeted implementation strategy on practitioner knowledge, practice and belief systems and to identify factors that predicted learning. The study was set in educational venues (conference rooms) in Sydney Australia and was approved by the Human Research Ethics Committee at the University of Sydney.

### Development of the implementation strategy

The implementation strategy was developed by the authors, the guideline developers and representatives from the target markets (the ‘working party’). Key target markets for guideline implementation were determined as: allied health professionals (physiotherapists, chiropractors and osteopaths), general practitioners, insurance personnel and consumers (people with whiplash). Different versions of the guidelines were developed for each of these target markets [see maa.nsw.gov.au] and specific strategies developed accordingly (eg [19]). This paper evaluates the effect of the strategy developed for allied health professionals.

The broad implementation strategy chosen for health professionals was to provide interactive education using opinion leaders, given the positive effects demonstrated in our previous trial [18], as well as evidence from systematic reviews [4-6]. One key difference with the current strategy, was that education was provided to three different professions (physiotherapy, chiropractic and osteopathy). Opinion leaders from each health profession were therefore identified by the working party and approached to provide the education.

A second difference was that education was targeted at improving knowledge and clinical practice in relation to the four key messages that were identified by the working party (Table 1). The educational content was developed by the working party, with further input from focus groups conducted with each of the three professional groups. The four key messages identified were: appropriate assessment and classification of whiplash, appropriate measure of outcome, able to identify those with poor prognosis, provides primarily activating treatments.

**Table 1:** Key messages of the whiplash guidelines for allied health professionals

**Key message**	**Response consistent with guideline recommendations**	**Measured by**
**Appropriate assessment and classification of whiplash**	Classifies whiplash according to the Quebec Task Force (QTF) [20] 4 grade classification system	Response to question 2 on the record of clinical practice
**Appropriate measure of outcome**	Uses a validated measure of outcome for whiplash such as the Neck Disability Index or the Patient Specific Functional Scale.	Response to question 4 on the record of clinical practice
**Identification of those with poor prognosis**	Identifies predictors of poor prognosis including high initial pain and high initial disability.	Response to question 6 on the record of clinical practice
**Provides primarily activating treatments**	Provided active treatment guidelines including exercise and advice	Response to question 5 on the record of clinical practice

Examples of content taught in relation to each key message are given below:

1) *Appropriate assessment:* Included basic assessment of range of motion and palpation in order to classify whiplash, and advanced assessment such as cold and pressure sensitivity and assessment of motor function. 2) *Use of functional outcome measures:* included discussion on reliability and responsiveness of various outcome measures in whiplash order to measure progress 3) *Identification of patients with a poor prognosis:* included assessing high initial pain intensity and disability with validated instruments such as the Visual Analogue Pain Scale (VAS) and the Neck Disability Index in order to identify people who have a higher probability of not recovering; 4) *Providing activating information* as per recommendations in the guideline that every whiplash patient at the first consultation should receive treatments which are largely activating advice and exercises.

A third key difference with the current implementation strategy was the recruitment strategy chosen to ensure that a proportion of the participants would be non-compliant with guidelines at entry to the study. Our prior experience had revealed that health professionals who volunteer for studies are more likely to be compliant with guidelines [18], hence leaving minimal scope to improve learning or compliance.

The working party decided to measure compliance with guidelines for this study by evaluating responses to a standard record of clinical practice. This was believed by the authors to be the measure that would most accurately reflect actual clinical practice. The record of clinical practice is a standard form used to report assessment findings and treatment choices to compulsory third party insurers in Australia. Non-compliance with clinical guidelines was considered as failure to adhere to at least three of the four key messages outlined above. In order to increase the likelihood of including participants who may be non-compliant with guidelines, we set up two participant recruitment strategies as outlined below.

### Participants

Participants in this study comprised allied health professionals in Australia who most commonly manage whiplash: physiotherapists, chiropractors and osteopaths. They were recruited by one of two methods.

1) *Recruitment by insurers*. Personnel working for compulsory third party (CTP) insurers were asked to review past records of clinical practice submitted by health professionals to the insurer. If they considered the responses on these records to be non-compliant on at least three of the four key messages, they were asked to provide this list of health professionals to the MAA.

2) *Recruitment by advertisement*. A further group of participants was recruited through advertising on professional association websites and newsletters. These included the three targeted health professions: the Australian Physiotherapy Association, the Chiropractor’s Association of Australia and the Australian Osteopathic Association.

The MAA then invited the health professionals identified by both methods to participate in the study. Once the health professional agreed to participate, their contact details were provided to the authors.

### Baseline assessment

Prior to attending education, all participants were assessed at baseline for professional knowledge, professional practice/ compliance with clinical guidelines and beliefs.

#### Professional knowledge

Professional knowledge was evaluated using a questionnaire adapted from that used by Rebbeck et al. [18]. Questions tested participant’s knowledge of each of the four key messages. The score range was from 0 (knowledge minimal and inconsistent with guidelines) to 39 (knowledge maximal and consistent with guidelines).

#### Professional practice/ compliance

Professional practice and compliance with clinical guidelines was evaluated by responses to a recent record of clinical practice. Each participant submitted a recent (within the past month) record of clinical practice for a patient with whiplash assessed by them. Responses recorded on the recent clinical record were then assessed independently by two authors (TR and LM) and categorized as compliant or non-compliant with guideline recommendations against each of the four criteria (Table 1). A participant was then classified at baseline as overall compliant with clinical guidelines if their answers were consistent with at least three of the criteria. Differences were resolved by consensus between the two authors. This method ensured baseline compliance was assessed consistently.

#### Beliefs

Beliefs of participating professionals were assessed using an adapted version of the Pain Attitudes and Beliefs Scale for Physiotherapists (PABS) [11]. The questionnaire was adapted by replacing the words ‘low back pain’ with ‘whiplash’. The PABS questionnaire gives a score for biomedical beliefs (ranging from 14 to 84) and a score for behavioural beliefs (ranging from 6 to 36).

### Intervention

The intervention was a two day interactive continuing educational workshop. Two workshops were offered within a six month period to cater for participant availability. Educational content was delivered by research and clinical opinion leaders in whiplash in Australia.

### Outcomes

Primary outcomes assessed were change in professional knowledge and professional practice. The secondary outcome was change in health professional’s beliefs. All outcomes were assessed at baseline and at three months after completion of the intervention.

#### Predictors of learning

The second aim of this study was to identify variables that may predict learning. Learning was defined as improvement in knowledge calculated as post knowledge questionnaire score – baseline knowledge questionnaire score. Predictor variables included demographic information (such as age, years of experience, profession and higher degree qualification) and the baseline measures such as compliance classification (compliant vs non compliant), biomedical belief score, behavioural belief score, and knowledge score. Lastly we used a measure of health professional confidence in their knowledge in our prediction model. This was assessed by asking health professionals how confident they felt in their knowledge related to three key messages in the guidelines (confidence in classifying patients, in predicting recovery and in treating patients). The confidence measure had been found to be related to compliance amongst general practitioners, and was being used in a concurrent study [19]. We were interested to see if this would also be a factor with other health professionals. Confidence was rated on a scale from 1 (don’t know) to 6 (very confident). The score range was 3 (minimal confidence) to 18 (maximum confidence).

### Statistical analyses

Data were analysed using PASW statistics for windows version 18.

Changes in continuous outcomes were assessed using ANOVA comparing baseline with post intervention scores and groups.

A McNemar’s test was used to assess changes in professional practice by comparing the number of professionals considered compliant with clinical guidelines at baseline with the number considered compliant post intervention.

Predictors of learning were analysed using multiple linear regression. A univariate linear regression was conducted to evaluate univariate associations of each one of the predictors of learning. All variables with p values greater than 0.25 in the univariate analyses were included in the multivariate model. A Backward elimination procedure was used (excluding non-significant predictors, p < 0.05) until only significant predictors remained in the model.

## Results

### Participants

**Figure 1:**
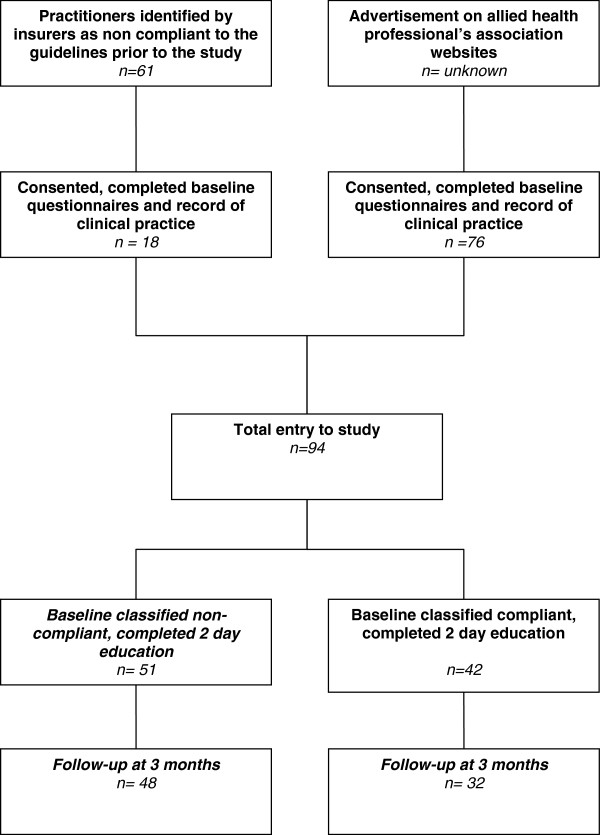
Flow chart of participants throughout the study.

Sixty one practitioners were identified by the insurers as likely to be non-compliant prior to entry in the study. Of these, 18 consented to participate in the study and completed baseline data. Seventy six practitioners were recruited from website or newsletter advertisement and consented to participate. Of these, one did not attend the workshop due to illness and was excluded from the study. From the total of 93 practitioners who joined our study, 51 were classified as non-compliant and 42 classified as compliant with the whiplash guidelines according to their responses to the recent baseline record of clinical practice provided to the authors. A total of 80 practitioners completed three- month follow up questionnaires, providing an 86% follow up rate. Reasons for loss to follow-up include: inability to contact (seven practitioners) and lack of time to respond to questionnaires (six practitioners). Figure 1 represents a flowchart for the study.

The demographic details for the participants at baseline are outlined in Table 2. A chi-square test demonstrated that there was a higher proportion of chiropractors in the non-compliant group when compared to the compliant group (p = 0.03). A significantly higher proportion of health professionals had post-graduate qualifications in the non-compliant compared with the compliant groups (p = 0.02). The knowledge score was also significantly different between groups (p = 0.03) with higher knowledge for compliant professionals. All other demographic characteristics were not significantly different between compliant and non-compliant participants.

**Table 2:** Baseline demographic details for participants in each classification

**Factor**	**Complaint at baseline N = 51**	**Non-complaint at baseline N = 42**
**Age: Mean(SD)**	37.2 (9.5)	36.2 (9.8)
**Years of experience**	13.2 (9.5)	11.5 (9.3)
**Profession (n/ %)**		
**Physiotherapists**	42 (82%)	25 (60%)
**Chiropractors**	9 (18%)	15 (36%)
**Osteopaths**	0 (0%)	2 (4%)
**Qualification (n/%)**		
**PhD**	1 (2%)	1 (3%)
**Masters**	13 (25%)	22 (52%)
**Bachelor**	37 (73%)	19 (45%)
**Knowledge score (0 to 39) Mean (SD)**	20.0 (5.7)	17.6 (4.6)
**Biomedical score (14 to 84)**	41.3 (7.6)	43.2 (8.9)
**Behavioural score (6 to 36)**	19.8 (3.4)	19.6 (4.3)
**Confidence score (3 to 18) Mean (SD)**	11.2 (2.9)	10.8 (2.4)

### Change in professional knowledge, practice and belief systems

There was a statistically significant difference between the baseline and post knowledge questionnaires (p < 0.0001; Table 3). Participants also significantly reduced their biomedical belief orientation (p = 0.000) and significantly increased their behavioural belief orientation (p = 0.02; Table 3).

**Table 3:** Baseline and post interventions scores for professional’s knowledge, beliefs and practice

**Factor**	**Baseline**	**Post intervention**	**Significance**
**Knowledge (0 to 39) Mean (SD)**	19.3 (5.3)	24.4 (5.3)	P = 0.000
**Biomedical belief (14 to 84)**	42.0 (8.3)	37.0 (8.3)	P = 0.000
**Behavioural belief (6 to 36)**	19.9 (3.8)	21.1 (4.1)	P = 0.02
**Professional practice**			
**% compliant**	58%	79%	P = 0.002
**1. Give WAD classification**	7%	41%	P < 0.0001
**2. Use appropriate measure of outcome**	77%	88%	P = 0.06
**3. Identifies those with poor prognosis**	15%	24%	P = 0.167
**4. Provides primarily activating treatments**	69%	74%	P = 0.63

Twenty one percent more participants were considered compliant with guidelines post intervention (79%) compared with (58%) at baseline (Table 3). This difference was statistically significant (p = .002). Of the four key criteria against which compliance was measured, participants improved their compliance significantly with the ability to classify whiplash (p < .0001) and did not improve with any other individual area. It was noted that compliance with two of the criteria (using functional outcome measures) and using activating treatments was already high at baseline (77% and 69% respectively).

We conducted further analysis on the third criteria against which compliance was assessed, namely ‘identification of those with poor prognosis’ as noted on the record of clinical practice. According to this record, the percentage of health professionals who were able to identify patients with a poor prognosis was low at baseline (15%) and did not significantly improve (24%). However health professionals only recorded prognostic factors on the record of clinical practice if they were present for the patient assessed. Therefore this measure may not be sensitive to actual knowledge regarding adverse prognostic factors. Therefore, in order to further examine whether practitioners did improve their knowledge on the identification of adverse prognostic factors, we further analysed an open ended question on the knowledge questionnaire, which asks “What features in a whiplash patient signal to you that a patient may not recover”. Health professionals would nominate factors they thought related to non-recovery and the researchers would then classify the responses into the categories nominated in the guidelines. The results indicated that significantly more health professionals were able to identify the main factors relating to non-recovery after the workshop (Table 4).

**Table 4:** Percentage of health professionals able to identify factors related to non-recovery at baseline and post-intervention

**Factor**	**Baseline %**	**Post intervention %**	**Sig (McNemar)**
High initial pain intensity^1^	35.1	61.7	<0.0001
High initial disability^1^	20.2	55.3	<0.0001
Demographic factors^2^	8.5	18.1	0.180
Crash related factors^2^	7.4	9.6	1
Radiographic factors^2^	5.3	1.1	0.375
Prior history^2^	16.0	5.3	0.453
Compensation factors^2^	8.5	8.5	1
Psychological distress^2^	45.7	33.0	0.486

^1^Considered an adverse prognostic indicator in the guidelines. Note that high initial pain intensity could be measured by a Visual Analogue Scale *(VAS)* and high initial disability by any of the recommended disability measures in the guidelines, such as the Neck Disability Index.

^2^Not considered an adverse prognostic indicator in the guidelines.

### Predictors of learning

A univariate linear regression was performed in order to identify variables that may predict learning (Table 5). The variables profession, biomedical score (baseline), baseline knowledge score, and baseline confidence had p values greater than 0.25 and were included in the multivariate model. A backward elimination was initially performed, and all variables that were not statistically significant in the model were excluded. The final model included profession and baseline knowledge questionnaire scores. This model explained 35% of the variance in learning (Adjusted R^2^). Specifically the data can be interpreted as participants with lower knowledge at baseline, learnt more. Each unit decrease in baseline knowledge, was associated with an 0.6 unit increase in learning. Similarly physiotherapists were associated with a 3.8 point unit increase in learning compared with non-physiotherapists.

**Table 5:** Univariate and multivariate models for predicting learning (post knowledge score – baseline knowledge score) related to whiplash clinical guidelines

**Variable**	**Effect estimates**	**Adjusted R-square**	**P value**
**Univariate analysis**			
Age	0.003	−0.01	0.96
Profession	−2.29	0.02	0.12
Years of experience	−0.04	−0.009	0.58
Qualification	−1.04	−0.005	0.44
Biomedical score (baseline)	0.11	0.01	0.17
Behavioural score (baseline)	0.13	−0.006	0.43
Baseline compliance	1.17	−0.002	0.37
Baseline knowledge score	−0.57	0.27	0.000
Baseline confidence	−0.32	0.01	0.16
**Final Model: Multivariate analysis**			
Profession	−3.8	0.35	0.003*
Baseline knowledge score	−0.6		0.000*

Direction of effect estimates for dichotomous outcomes. ***Profession***, Negative effect estimates indicate that non-physiotherapists learned less than other professionals. ***Qualification***, Negative effect estimates indicate that professionals with a graduate degree learnt less than those with a bachelor degree. ***Baseline compliance***, Positive effect estimates indicate that participants that were compliant with guidelines learned more than those that were not compliant at baseline.

## Discussion

Following a targeted implementation strategy there were large improvements in health professional’s knowledge of whiplash management, beliefs about pain and clinical practice so that it aligned more closely with recommendations in guidelines for whiplash. As hypothesised, knowledge improvement was greatest in practitioners with low baseline knowledge and those whose professional background was physiotherapy. This information may be useful for organisations that implement guidelines, suggesting that educational resources may be best directed at practitioners whose knowledge of the guidelines is poor at baseline. It remains to be tested, whether this strategy would in turn result in greater health outcomes for patients with whiplash.

The improvement in knowledge and clinical practice after the active implementation strategy used in this study would be considered large, when considering previously published effect sizes. It should be remembered that this study in not an RCT, so direct comparisons of effect sizes cannot be made. However, the aim of this study was to prove a concept, namely that those with poor baseline knowledge and compliance would learn more. The proven concept was that low baseline knowledge significantly contributed to the size of the learning effect. Identifying such practitioners is practical in whiplash, because it is a condition primarily arising from a motor vehicle accident and treatment is commonly regulated by insurers, where information regarding health professional knowledge about guidelines can be easily obtained. The results of this study therefore have implications for organisations implementing guidelines for whiplash. They suggest that by measuring baseline knowledge through a questionnaire, implementation costs could be saved by directing education at practitioners with low knowledge, rather than attempting to educate all practitioners.

A key difference in this study to the previous RCT conducted by the authors [18] was the recruitment strategy, aimed to include practitioners whose baseline compliance with guidelines was low. The current strategy appeared effective, given that a considerable proportion (46%) of our participants were assessed as non-compliant at baseline. We hypothesised, however, that non-compliance at baseline would be a predictor of learning, and did not find this. Explanations for this may be statistical, namely we classified people dichotomously as compliant or non-compliant, rather than using a continuous variable that may be more responsive or predictive, as suggested by systematic reviewers [4]. Alternatively, it may be that other factors are more predictive of outcome, such as baseline knowledge and professional background proved to be in this study. In Forsetlund et al's (2009) systematic review, which reviewed the effect of interventions most like ours, significant effect modifiers included the intensity of the education, attendance at education, complexity of the targeted behaviours and seriousness of the outcome. The interpretation of our results together with that of Forsetlund et al (2009) would suggest that known effect modifiers should be considered in future implementation strategies, and that measures of baseline compliance should be a continuous rather than dichotomous variable.

Barriers to implementation have been identified and addressed in order to improve compliance with guidelines in the medical field [21,22]. However, only recently have data been available for allied health professions such as physiotherapy in terms of implementation strategies. Authors of these studies suggest that clinicians use guidelines if recommendations are similar to their usual practices [14] and beliefs [12] or they can perceive an advantage from using them [17] and note deviation from guidelines if practitioners have little experience with managing the condition [15]. However to date such factors have only accounted for a small percentage of the variance in predicting guideline adherence. For example practitioner factors including perceived advantage accounted for 5.6% of the variance in a model to explain determinants of guideline adherence by physiotherapists to the Dutch guidelines for LBP [17]. The two factors found to predict knowledge to be more consistent with guideline recommendations in our study were low baseline knowledge and profession, accounting for 35% of the variance. Because these data predict a high variance, they suggest that our identified factors should be considered when designing future implementation strategies.

The finding that profession predicted learning in our study warrants further discussion. We found that physiotherapists learned more than chiropractors or osteopaths. These results may reflect the fact that opinion leaders providing the education program were mainly physiotherapists and could have added their experiences and perspectives to the program. However, the content of the clinical guidelines advocate more active (exercise) approaches that may be more familiar to physiotherapists than chiropractors. Recent debates have suggested that if practitioners have more biomedical beliefs that they are less likely to advocate for an active approach than practitioners that have more behavioural beliefs [11,12]. Therefore, we measured the beliefs of practitioners using the PABS [11] questionnaire, but did not find beliefs to be a predictor of learning. However when we compared beliefs between practitioners, we found that whilst behavioural orientation was similar between professionals, chiropractors and osteopaths had statistically higher biomedical scores than physiotherapists (p = 0.01) which may explain our data. This finding is similar to that reported by Pincus et al. [13] where chiropractors were found to have a more biomedical approach to managing low back pain than physiotherapists [13]. Implementation scientists therefore may need to consider professions and their beliefs when developing an implementation strategy.

## Conclusions

In conclusion, factors including low baseline knowledge and profession should be considered when designing future implementation strategies for whiplash guidelines. Based on these data, it is recommended to target the strategy to the identified gaps in the knowledge and to the specific profession. It is hypothesised and remains to be tested as to whether such a strategy will in turn improve health outcomes for patients with whiplash.

## Competing interests

This study was funded by the Motor Accidents Authority (MAA) of New South Wales Australia.

## Authors’ contributions

TR conceived and designed the study, co-ordinated and provided part of the education, assisted in data collection and drafted the manuscript. LM assisted in data collection, performed the statistical analysis and interpretation of data and was involved in drafting and revising the manuscript. CM assisted in study design, data analysis and in revising the manuscript. All authors read and approved the final manuscript.

## Pre-publication history

The pre-publication history for this paper can be accessed here:

http://www.biomedcentral.com/1472-6963/13/213/prepub
